# Supplementary material for: Chromatin accessibility landscape of relapsed pediatric B-lineage acute lymphoblastic leukemia
Source: Nat Commun. 2023 Oct 25;14:6792. doi: 10.1038/s41467-023-42565-z (PMC10600232; doi:10.1038/s41467-023-42565-z)
Supplement: Supplementary file 1 — Supplementary Information [file 41467_2023_42565_MOESM1_ESM.pdf]

Supplementary Figures for

Chromatin accessibility landscape of relapsed pediatric B-lineage acute lymphoblastic leukemia

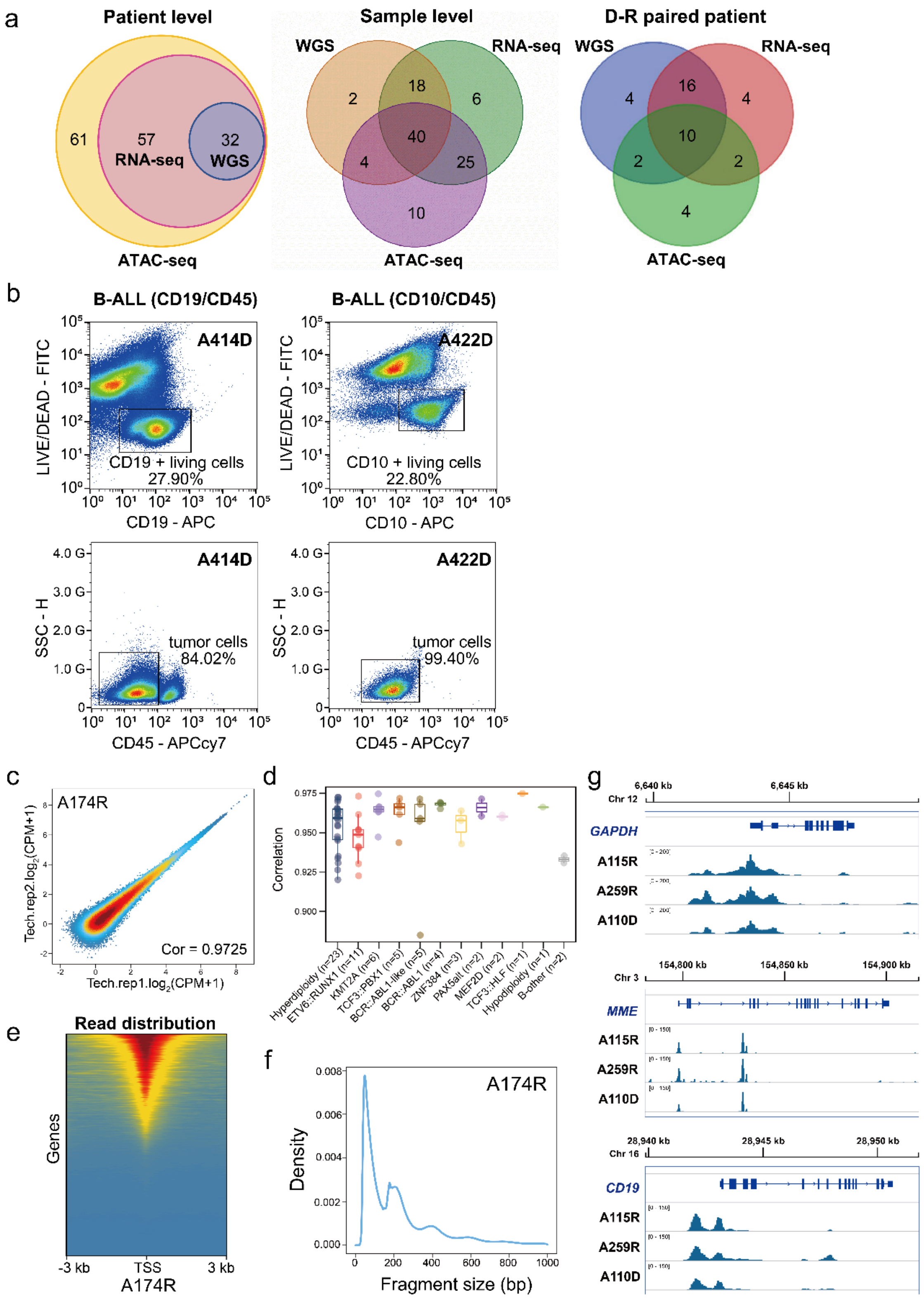

Supplementary Figure 1. Quality control of ATAC-seq data. (a) Venn diagram showing the number of WGS, RNA-seq, and ATAC-seq data sets analyzed in this study at the patient level (left) and sample level (middle). Venn diagram on the right shows the number of diagnosis (D)-relapse (R) paired samples analyzed in each experiment. (b) Purification of tumor cells by flow cytometry. The antibodies used for A414D and A422D are indicated

above the plots. (c) Scatterplot showing the reproducibility of ATAC-seq data between two technical replicates of a representative sample, A174R. Each dot represents an individual ACR. (d) Box plots showing the Pearson correlation coefficients between all technical replicates for samples (n = 65) in each subtype. Box plots show the median number as centers, the upper and lower hinges represent 75th and 25th percentile, and whiskers extend to largest and smallest values no more than 1.5*IQR. (e) Enrichment of ATAC-seq accessibility signals near the transcription start site (TSS ± 3000 bp) in A174R. (f) Distribution of Tn5 insertion fragment sizes for A174R. (g) Normalized ATAC-seq profiles showing chromatin accessibility in the representative gene regions *GAPDH*, *MME*, and *CD19*. Y axis scale ranges from 0 - 200 in normalized units.

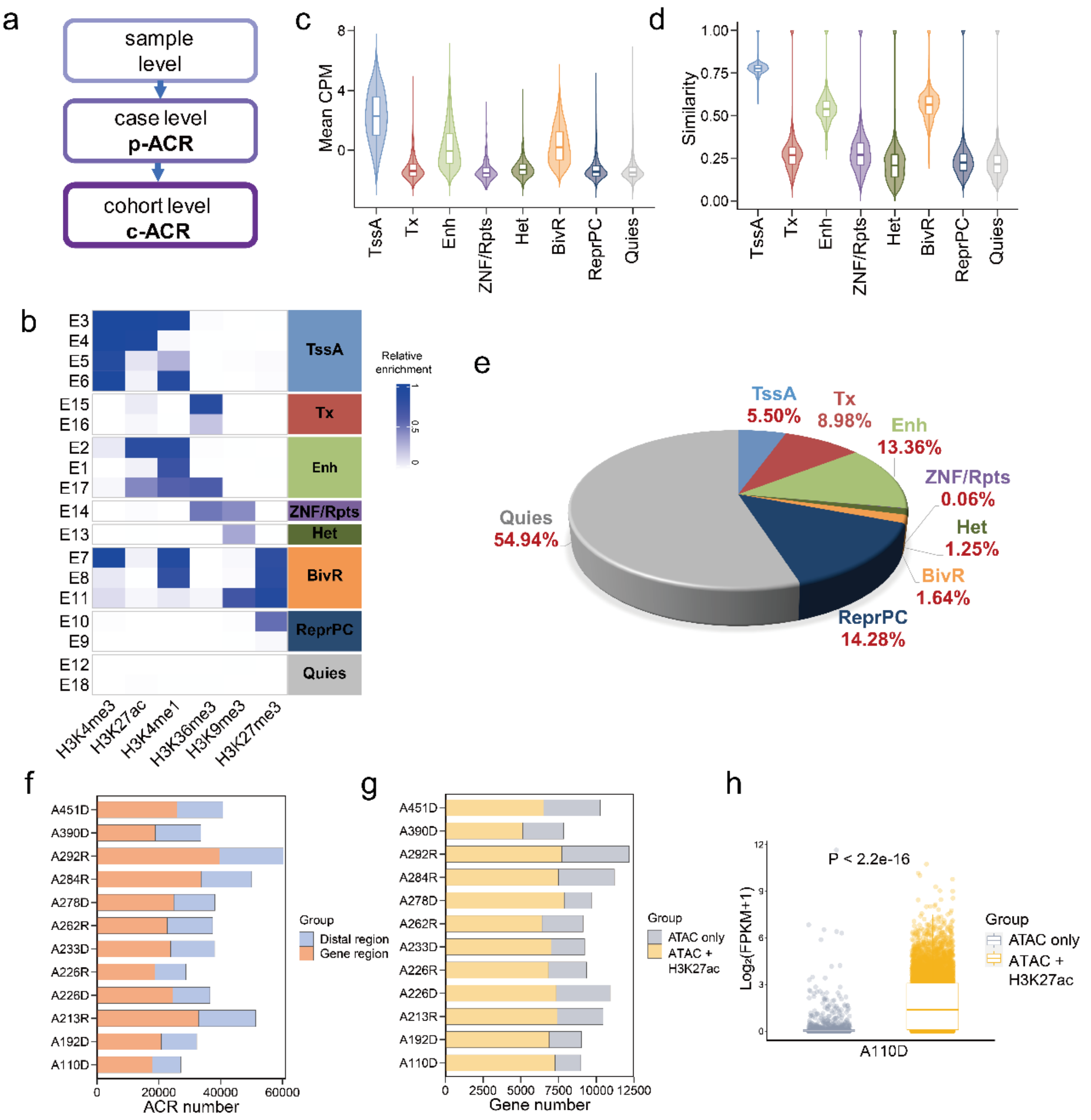

Supplementary Figure 2. Characteristics of chromatin accessibility in different genomic regions. (a) Diagram showing ACR data sets at different levels. p-ACR, patient level ACR set. c-ACR, cohort level ACR set. (b) Heatmap showing the genomic states classification based on ChIP-seq data of histone modifications in primary B-ALL by ChromHMM algorithm. (c) The mean chromatin accessibility of ACRs residing within different genomic regions across all 75 B-ALL samples. CPM, counts per million. Box plots show the median number as centers, the upper and lower hinges represent 75th and 25th percentile, and whiskers extend to largest and smallest values no more than 1.5*IQR. (d) The similarity of ACRs between samples for different genomic regions. A total of 758,738 c-ACRs from 75 samples were included in this analysis. For each pair of samples, the similarity was calculated as the number of overlapping ACRs divided by total number of unique ACRs. Box plots show the median number as centers, the upper and lower hinges represent 75th and 25th percentile, and whiskers extend to largest and smallest values no

more than 1.5*IQR. (e) Pie chart showing the proportion of 758,738 c-ACRs mapping to different genomic regions. (f) Bar plots shows the association between Quies ACRs and gene regions. Quies ACRs located in gene regions (by extending of 5% length of the gene sequences up- and down-stream of gene) are shown in red. The remaining ACRs are classified as being located in distal regions and shown in blue. Each line represents one B-ALL sample. (g) Bar plots presenting the proportions of genes intersected with Quies ACRs and H3K27ac signals (ATAC + H3K27ac) or with Quies ACRs only (ATAC only). Each line represents one B-ALL sample. (h) The RNA expression levels of genes in the neighborhood of Quies ACRs that overlap H3K27ac modifications (ATAC + H3K27ac) or not (ATAC only) in A110D. Two-sided Wilcoxon test, $P < 2.2e\text{-}16$.

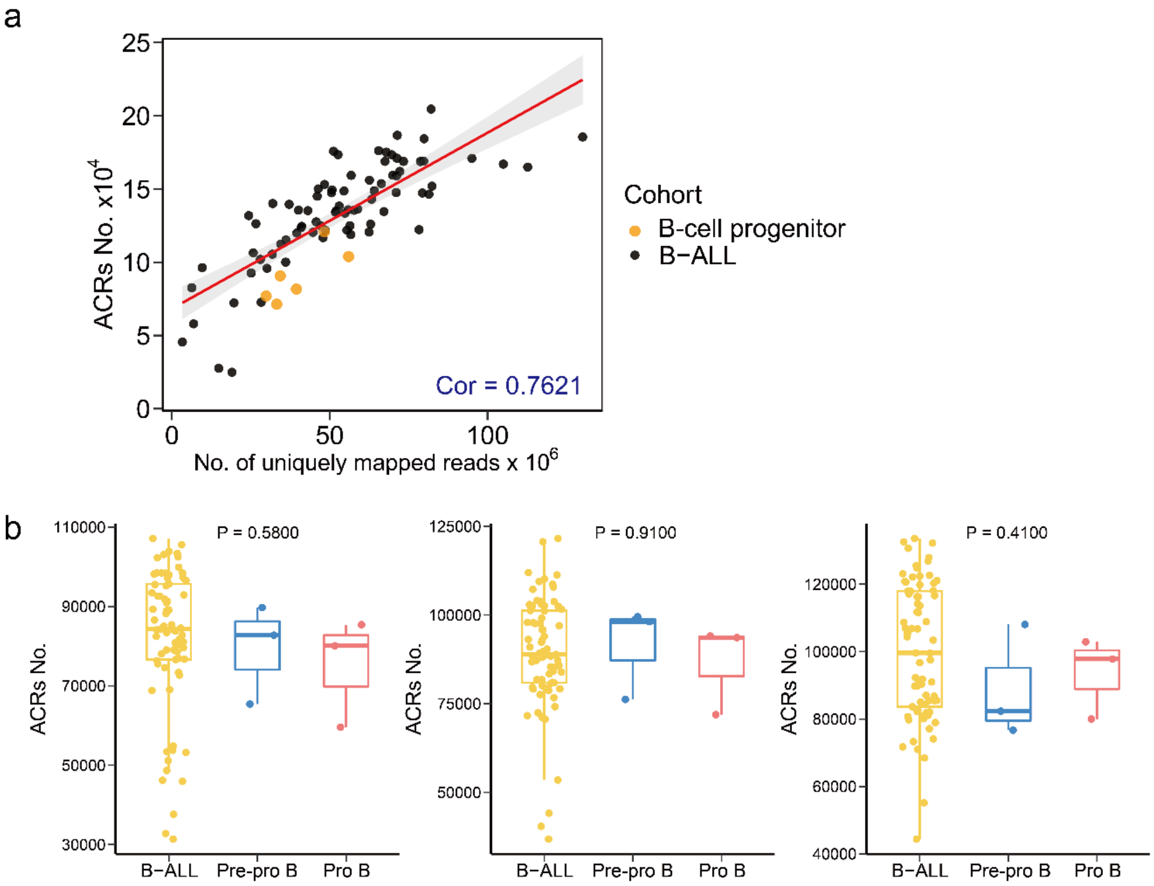

Supplementary Figure 3. Comparison of open chromatin between B-cell progenitors and B-ALLs. (a) Correlation between the number of uniquely mapped reads and the number of ACRs detected for B progenitor cells (3 pre-pro B cells and 3 pro B cells) using data published previously[1] (in yellow) and data for 75 B-ALL samples generated in this study (in black). (b) Differences in accessible chromatin regions between B progenitor cells and B-ALLs (B-ALL n = 75, Pre-pro B n = 3, Pro B n =3). For each sample, data were down sampled to twenty million (left), thirty million (middle) and forty million (right) reads for comparison. Two-sided Wilcoxon test, P values are labeled in each plot. Box plots show the median number as centers, the upper and lower hinges represent 75th and 25th percentile, and whiskers extend to largest and smallest values no more than 1.5*IQR.

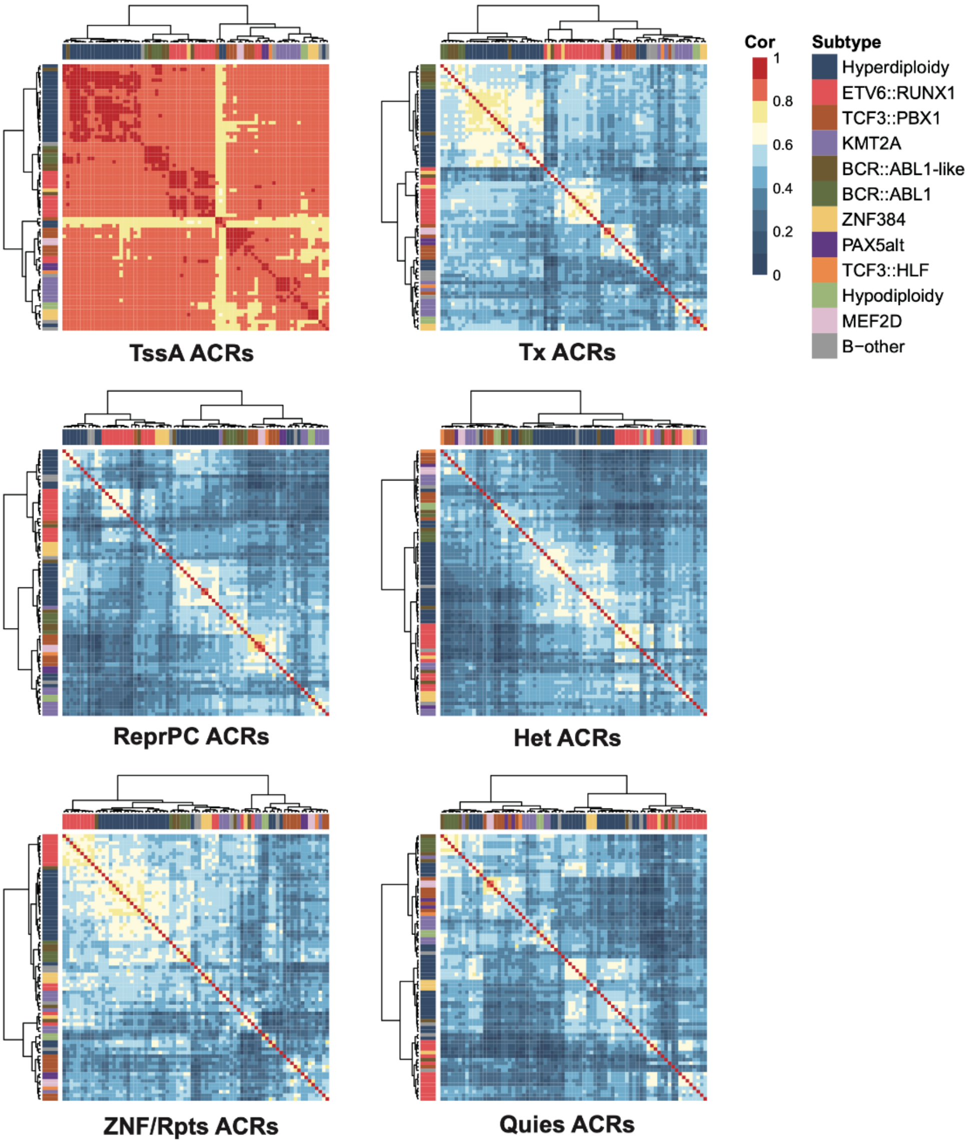

Supplementary Figure 4. Pairwise sample similarity of chromatin accessibility regions. Heatmaps showing the pairwise Pearson correlation coefficients between samples based on accessibility of ACRs. The ACRs were separated according to the six different functional genomic regions, namely TssA, Tx, ReprPC, Het, ZNF/Rpts, and Quies. The correlations were analyzed among ACRs in each individual type of functional genomic regions. Color scale values are consistent between heatmaps.

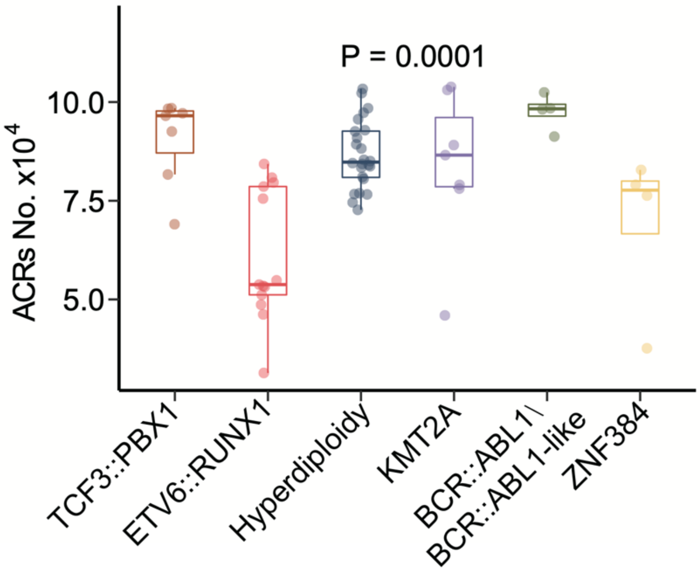

Supplementary Figure 5. The number of chromatin accessibility regions for each subtype in B-ALL. The numbers of ACRs in each subtype are shown in boxplots. Each dot represents one individual sample (TCF3::PBX1 n = 7, ETV6::RUNX1 n = 13, Hyperdiploidy n = 24, KMT2A n = 7, BCR::ABL1\BCR::ABL1-like n = 4, ZNF384 n = 4). Data were down sampled to twenty million reads for comparison (P = 0.0001, Kruskal-Wallis test). Box plots show the median number as centers, the upper and lower hinges represent 75th and 25th percentile, and whiskers extend to largest and smallest values no more than 1.5*IQR.

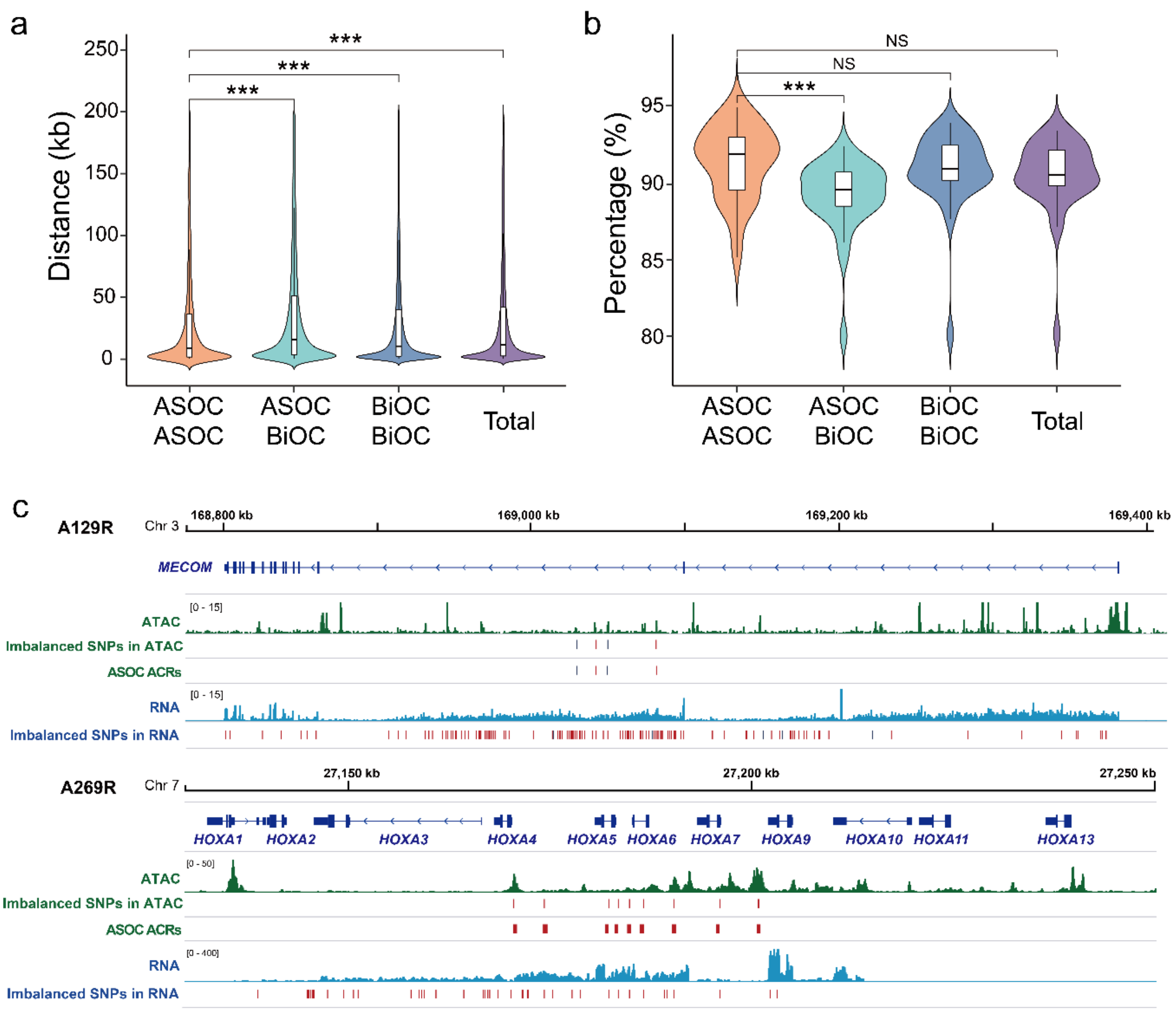

Supplementary Figure 6. The characteristics of allele-specific open chromatin regions in B-ALL. (a) The distance between two adjacent ACRs for different ACR pairs. (Two-sided Wilcoxon test, ***, P < 0.0001; ASOC-ASOC vs ASOC-BiOC P < 2.2e-16, ASOC-ASOC vs BiOC -BiOC P < 2.2e-16, ASOC-ASOC vs Total P < 2.2e-16). Box plots show the median number as centers, the upper and lower hinges represent 75th and 25th percentile, and whiskers extend to largest and smallest values no more than 1.5*IQR. (b) The percentage of adjacent ACRs grouped in a single topological associated domain. (Two-sided Wilcoxon test, ***, P < 0.0001; ASOC-ASOC vs ASOC-BiOC P = 0.0002, ASOC-ASOC vs BiOC-BiOC P = 0.3890, ASOC-ASOC vs Total P = 0.1460). Box plots show the median number as centers, the upper and lower hinges represent 75th and 25th percentile, and whiskers extend to largest and smallest values no more than 1.5*IQR. A total of 32 samples were included in the analysis in (a) and (b), with one sample analyzed for each patient. Diagnosis sample was included for the patient with paired diagnosis and relapsed tumor samples. For patient with only diagnosis or relapsed tumor sample, the available sample was analyzed. (c) Wiggle plots showing the allele-specific expression and allele-specific open chromatin of representative COSMIC genes. *MECOM* in A129R is shown in the upper panel, and HOXA

cluster genes in A269R are shown at the bottom. Heterozygous variants from WGS are labeled below. Markers showing imbalanced chromatin accessibility and ASOC ACRs are shown in red, and biallelic markers and BiOC regions are colored in blue.

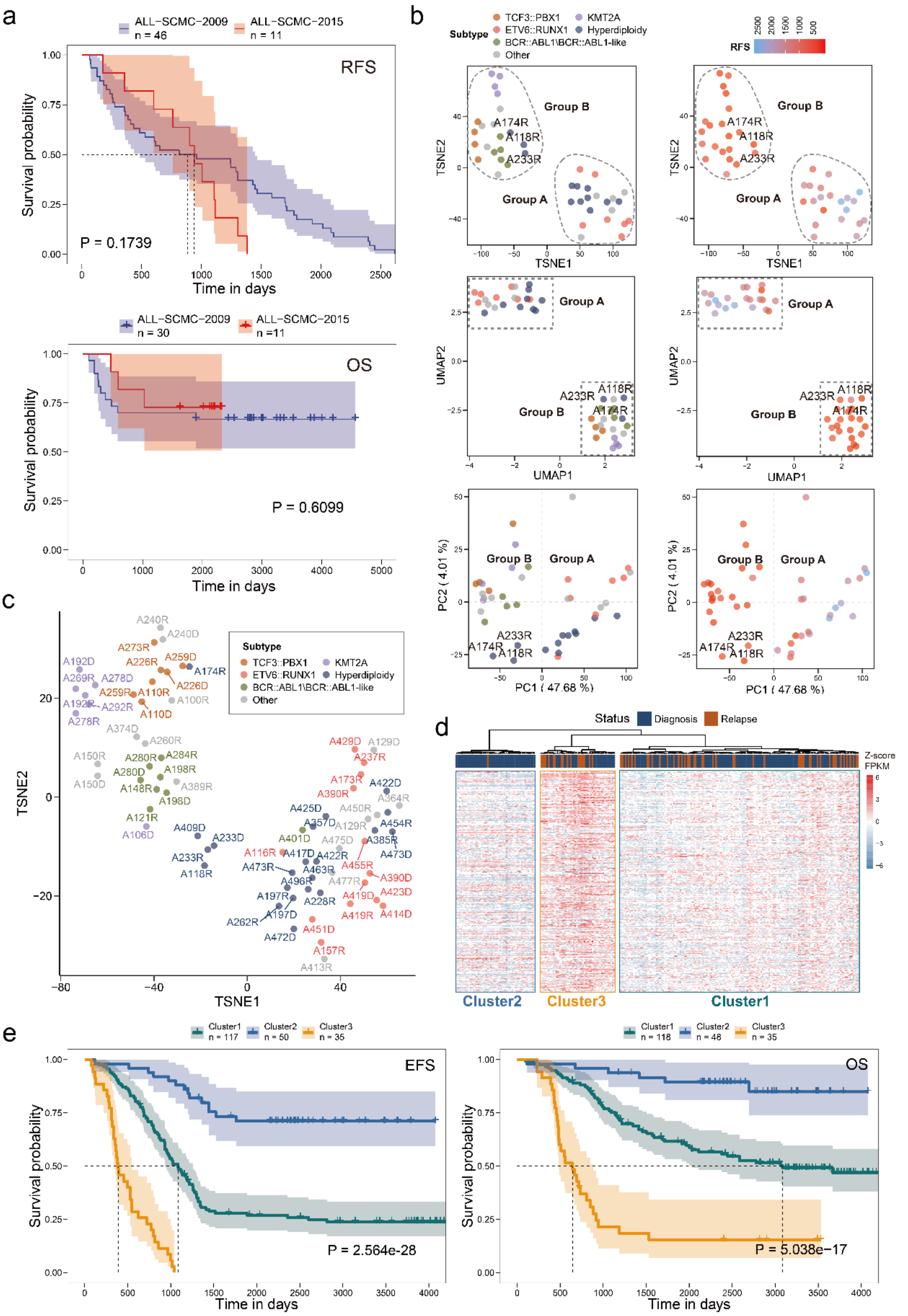

Supplementary Figure 7. Chromatin accessibility is associated with the prognoses of B-ALL patients. (a) RFS (up, 57 patients) and OS (down, 41 patients) were estimates for B-ALL patients with sufficient follow-up information. No significant difference was observed between patients treated with the ALL-SCMC-2009 and ALL-SCMC-2015 protocols (Log-

rank test). The error bands indicate 95% confidence intervals, and the dash line indicate the median survival time. (b) The unsupervised clustering with 3 methods (t-SNE, UMAP and PCA) of 42 relapse B-ALL cases based on the top 10% most significant RFS-related ACRs. Each dot represents an individual sample. The color codes represent for subtype and the time to relapse for the plots on the left and right column respectively. (c) The t-SNE plot of 42 relapse and 29 diagnosis samples based on the same RFS-related ACRs shown in Figure 5b. Each dot represents an individual sample, and color represents B-ALL subtype. (d) Unsupervised clustering analysis of 252 B-ALL samples from the TARGET project based on the expression of target genes potentially regulated by differential ACRs with increased accessibility in B-ALLs of Group B. (e) Event-free survival rate (EFS, 202 patients) and overall survival rate (OS, 201 patients) estimates for B-ALL samples of three clusters presented in Supplementary Figure 7d. Only patients with sufficient follow-up information were included. For patients with paired diagnosis and relapsed samples, the patient was assigned to the cluster of relapsed samples in the survival analysis. The error bands indicate 95% confidence intervals, and the dash line indicate the median survival time. Log-rank test, EFS P = 2.564e-28 and OS P = 5.038e-17.

Supplementary References

1. O'Byrne S*, et al.* Discovery of a CD10-negative B-progenitor in human fetal life identifies unique ontogeny-related developmental programs. *Blood* **134**, 1059-1071 (2019).
