# Supplementary material for: Chromatin accessibility landscape of relapsed pediatric B-lineage acute lymphoblastic leukemia
Source: Nat Commun. 2023 Oct 25;14:6792. doi: 10.1038/s41467-023-42565-z (PMC10600232; doi:10.1038/s41467-023-42565-z)
Supplement: Supplementary file 3 — Description of Additional Supplementary Files [file 41467_2023_42565_MOESM3_ESM.pdf]

Description of Additional Supplementary Files

File Name: Supplementary Data 1
Description: **Information of clinic characteristics and data availability for 61 patients profiled in this study.** Data generated in this study were highlighted in red. Informed written consents were obtained from parents for all patients whose clinical information were listed below. P on columns L to O represents for published data. Column N and O listed the types of RNA-seq applied, with A and B stands for total stranded RNA-seq in two sequencing centers and C stands for mRNA-seq.The RNA-seq data with*(A423R.RNAseq(C)*) failed quality control and was excluded from further analysis. Column R shows the subtype-related lesions in WGS data, the Columns S to T represents for subtype-related fusions detected from RNA-seq data. Fusions labeled with * on Columns S to T represent for those that were rescued by reviewing bam files, and the number of reads supporting the fusion was showed in brackets. Column U and V diaplays the subtypes predicted from gene expression pattern from RNA-seq data. Column W shows the karotype of each patient from clinical test. Column X-AA represents for the copy number variations from analyzing RNA-seq data."-" stands for no data and "None" stands for no results.

File Name: Supplementary Data 2
Description: **The percentage of tumor cells positive for CD19 and/or CD10.** Columns B-F for diagnosis samples and columns G-K for relapsed samples. Columns C and H represent for the proportion of tumor cells from morphological examination before purification, and columns D and I represent for the proportion of tumor cells from flow cytometric immunophenotyping before purification. The ratio of CD19/CD10 positive tumor cells among all tumor cells were listed in column E/F/J/K. The antibody selected for purifying tumor cells for each samples was listed in column B and G.

File Name: Supplementary Data 3
Description: **Characteristics of sequencing statistics for 144 ATAC-seq data.** Prealignment reads were that align to the mitochondrial genome and human alpha satellite repeats, Alu repeats, ribosomal DNA repeats, and other repeat regions.

File Name: Supplementary Data 4
Description: **The genomic positions and annotations of the c-ACRs.**

File Name: Supplementary Data 5
Description: **The differential ACRs between B-ALL samples and B-cell progenitor cells.** "B-ALL_high" represents for 252,028 ACRs that are significantly higher in B-ALL. "B-ALL_low" represents for 26,045 ACRs that are significantly lower in B-ALL. The standard for differential ACRs is FDR < 0.05 & |Log2FoldChange| >1. P values were calculated by Wald test and adjusted for multiple testing using the procedure of Benjamini and Hochberg (by DESeq2).

File Name: Supplementary Data 6
Description: **The Biological Process enrichment results of 2,332 protein coding genes targeted by 252,028 differential ACRs with higher accessibility in B-ALL.** P values were calculated by one-sided Fisher test and adjusted by multiple testing by Benjamini-Hochberg FDR.

File Name: Supplementary Data 7
Description: **The list of 17,981 subtype-specific ACRs and their associated subtypes.**

File Name: Supplementary Data 8
Description: **The ASOC and BiOC regions identified in individual samples.** The significance of the imbalanced accessibility between two alleles for each ACR were listed on column F. Difference of chromatin accessibility between two alleles in each region were listed on column G (Methods). ASOC was defined as P value < 0.05 (Methods) and absolute delta >= 0.2, while other ACRs were grouped as BiOC. The ASOC status for each peak was listed on column H.

File Name: Supplementary Data 9
Description:
Supplementary Data 9A. 1,771 dysregulated ACRs in relapse in TCF3::PBX1 subtype. P values were calculated by Wald test and adjusted for multiple testing using the procedure of Benjamini and Hochberg (by DESeq2).
Supplementary Data 9B. 945 dysregulated ACRs in relapse in ETV6::RUNX1 subtype. P values were calculated by Wald test and adjusted for multiple testing using the procedure of Benjamini and Hochberg (by DESeq2).
Supplementary Data 9C. 4,072 dysregulated ACRs in relapse in BCR::ABL1\BCR::ABL1-like subtype. P values were calculated by Wald test and adjusted for multiple testing using the procedure of Benjamini and Hochberg (by DESeq2).
Supplementary Data 9D. 268 dysregulated ACRs in relapse in hyperdiploidy subtype. P values were calculated by Wald test and adjusted for multiple testing using the procedure of Benjamini and Hochberg (by DESeq2).
Supplementary Data 9E. 371 dysregulated ACRs in relapse in KMT2A subtype. P values were calculated by Wald test and adjusted for multiple testing using the procedure of Benjamini and Hochberg (by DESeq2).
Supplementary Data 9F. Combined relapse-high ACRs in 5 molecular subtypes. P values were calculated by Wald test and adjusted for multiple testing using the procedure of Benjamini and Hochberg(by DESeq2).
Supplementary Data 9G. Combined relapse-low ACRs in 5 molecular subtypes.

File Name: Supplementary Data 10
Description: **116,307 ACR-gene correlations identified by ACR-to-gene analysis.**

File Name: Supplementary Data 11
Description: **Target genes of differential ACRs dysregulated in relapse for each subtype predicted by ACR-to-gene analysis.**

File Name: Supplementary Data 12
Description: **Gene transcription associated to treatment response of eight clinical drugs used in ALL therapy.** P values were calculated by two-sided Fisher exact test.

File Name: Supplementary Data 13
Description:
Supplementary Data 13A. The 70,573 RFS-related ACRs identified by Log-rank test.
Supplementary Data S13B. The potential target genes of RFS-related ACRs predicted by ACR-to-gene

File Name: Supplementary Data 14
Description:

Supplementary Data 14A. 31,632 RFS-related ACRs with differential accessibility between B-ALL cases in Group A and Group B. P values were calculated by Wald test and adjusted for multiple testing using the procedure of Benjamini and Hochberg (by DESeq2).
Supplementary Data S14B. The potential target genes of RFS-related ACRs with higher chromatin accessibility in B-ALL cases in Group B, as predicted from ACR-to-gene links.

File Name: Supplementary Data 15
Description: **The clustering information of 252 B-ALL samples of TARGET corhort in Supplementary Figure 7D.**

File Name: Supplementary Data 16
Description:
Supplementary Data 16A. 7,566 differential ACRs between hyperdiploidy cases in Group A and Group B. P values were calculated by Wald test and adjusted for multiple testing using the procedure of Benjamini and Hochberg (by DESeq2).

Supplementary Data S16B. The potential target genes of differential ACRs with higher chromatin accessibility in hyperdiploidy cases in Group B, as predicted from ACR-to-gene links.
