# Supplementary material for: Chromatin accessibility landscape of relapsed pediatric B-lineage acute lymphoblastic leukemia
Source: Nat Commun. 2023 Oct 25;14:6792. doi: 10.1038/s41467-023-42565-z (PMC10600232; doi:10.1038/s41467-023-42565-z)
Supplement: Supplementary file 20 — Reporting Summary [file 41467_2023_42565_MOESM20_ESM.pdf]

nature portfolio

Corresponding author(s): Shuhong Shen and Yu Liu

Last updated by author(s): Oct 9, 2023

Reporting Summary

Nature Portfolio wishes to improve the reproducibility of the work that we publish. This form provides structure for consistency and transparency in reporting. For further information on Nature Portfolio policies, see our Editorial Policies and the Editorial Policy Checklist.

Statistics

For all statistical analyses, confirm that the following items are present in the figure legend, table legend, main text, or Methods section.

n/a	Confirmed	
☐	☒	The exact sample size (*n*) for each experimental group/condition, given as a discrete number and unit of measurement
☐	☒	A statement on whether measurements were taken from distinct samples or whether the same sample was measured repeatedly
☐	☒	The statistical test(s) used AND whether they are one- or two-sided<br>*Only common tests should be described solely by name; describe more complex techniques in the Methods section.*
☐	☒	A description of all covariates tested
☐	☒	A description of any assumptions or corrections, such as tests of normality and adjustment for multiple comparisons
☐	☒	A full description of the statistical parameters including central tendency (e.g. means) or other basic estimates (e.g. regression coefficient) AND variation (e.g. standard deviation) or associated estimates of uncertainty (e.g. confidence intervals)
☐	☒	For null hypothesis testing, the test statistic (e.g. *F*, *t*, *r*) with confidence intervals, effect sizes, degrees of freedom and *P* value noted<br>*Give P values as exact values whenever suitable.*
☒	☐	For Bayesian analysis, information on the choice of priors and Markov chain Monte Carlo settings
☒	☐	For hierarchical and complex designs, identification of the appropriate level for tests and full reporting of outcomes
☐	☒	Estimates of effect sizes (e.g. Cohen's *d*, Pearson's *r*), indicating how they were calculated

Our web collection on statistics for biologists contains articles on many of the points above.

Software and code

Policy information about availability of computer code

Data collection	No softwares were used for data collection.
Data analysis	Softwares used in this analysis include R (v4.0.2), Burrows-Wheeler Aligner (v0.7.17-r1188), FastQ Screen (v0.13.0), FastQC (v0.11.9), Bowtie2 (v2.4.1), SAMtools (v1.7), Picard (v2.22.9), MACS2 (v2.2.6), BEDTools (v2.29.2), edgeR (v3.32.1), FIMO (v 5.0.5), STAR (v2.7.1a), HTSeq count (v0.11.2), Matrix eQTL (v2.3), DESeq2 (v1.30.1) , RNAseqCNV (v1.2.2), Arriba (v2.1.0) and survival (v3.2-3).

For manuscripts utilizing custom algorithms or software that are central to the research but not yet described in published literature, software must be made available to editors and reviewers. We strongly encourage code deposition in a community repository (e.g. GitHub). See the Nature Portfolio guidelines for submitting code & software for further information.

Data

Policy information about availability of data

All manuscripts must include a data availability statement. This statement should provide the following information, where applicable:

- Accession codes, unique identifiers, or web links for publicly available datasets
- A description of any restrictions on data availability
- For clinical datasets or third party data, please ensure that the statement adheres to our policy

All analysis in this study use reference genome of human (hg19) [https://ftp.ncbi.nlm.nih.gov/genomes/archive/old_genbank/Eukaryotes/vertebrates_mammals/Homo_sapiens/GRCh37/special_requests/]. ATAC-seq, RNA-seq and ChIP-seq data generated in this study have been deposited in the Genome Sequence Archive

for Human (GSA-human) of the National Genomics Data Center of China under accession number HRA002815 [https://ngdc.cncb.ac.cn/gsa-human/browse/HRA002815]. The data is available for academic use under controlled access in compliance with the regulation of the Ministry of Science and Technology (MOST) of China for the deposit and use of human genomic data. Access can be obtained by contacting members of the Data Access Committee (DAC) following the application procedure in GSA. For detailed guidance, see GSA-Human_Request_Guide_for_Users [https://ngdc.cncb.ac.cn/gsa-human/document/GSA-Human_Request_Guide_for_Users_us.pdf]. Data will be available immediately once the application was approved. The access to the controlled data will be valid for one year from the date approved. The WGS data for 32 B-ALL patients and RNA-seq data for 29 B-ALLs were collected from previously published data (PMID: 31697823). Among these published data, the RNA-seq data were available in GSA-human of the National Genomics Data Center of China under accession number HRA000119 [https://ngdc.cncb.ac.cn/gsa-human/browse/HRA000119], the processed genomic alterations from WGS data were obtained from authors of the published paper (PMID: 31697823) with raw data available in GSA-human under accession number HRA005668 [https://ngdc.cncb.ac.cn/gsa-human/browse/HRA005668]. The publicly available ATAC-seq data of 3 pre-pro B cells and 3 pro B cells were available in the National Center for Biotechnology Information's Gene Expression Omnibus with accession number GSE122989 [https://www.ncbi.nlm.nih.gov/geo/query/acc.cgi?acc=GSE122989]. The hyperdiploidy B-ALL cases of TARGET dataset were downloaded from Target website (dbGaP Sub-study ID phs000464) [https://gdc.cancer.gov/about-data/publications#/?groups=TARGET-ALL-P2&years=&order=desc]. Only 43 samples with definitive molecular evidence for hyperdiploidy subtype were included in this analysis from TARGET dataset. ChIP-seq data of 6 histone modification markers (H3K4me1, H3K4me3, H3K9me3, H3K27ac, H3K27me3 and H3K36me3) were collected from Blueprint Epigenome Consortium (Donor ID: S017E3) [https://epigenomesportal.ca/ihec/grid.html?build=2020-10&assembly=4&institutions=3] and corresponding input raw data were downloaded from EGA database under accession number EGAD00001002421[https://ega-archive.org/datasets/EGAD00001002421]. The RNA expression data (DepMap Public 21Q1) and drug responses (Drug sensitivity AUC (CTD^2)) of 11 B-ALL cell lines were downloaded from DepMap database [https://depmap.org/portal/download]. The COSMIC genes (release v87) were download from COSMIC database [https://cancer.sanger.ac.uk/cosmic/download]. The remaining data are available within the Article and Source Data files. Source data are provided with this paper.

Research involving human participants, their data, or biological material

Policy information about studies with human participants or human data. See also policy information about sex, gender (identity/presentation), and sexual orientation and race, ethnicity and racism.

Reporting on sex and gender	The gender is not considered in study design. We did not report the gender of patients in this study.
Reporting on race, ethnicity, or other socially relevant groupings	We did not include these information in this study.
Population characteristics	61 relapsed B-ALL patients treated in Shanghai Children's Medical Center through 2007–2019 were included. 17 patients was diagnosed under age of 3, 30 patients between age 3-10, and 14 patients from 10-15.
Recruitment	Patients involved in this study were treated in Shanghai Children's Medical Center, Shanghai, China. All the relapsed patients with adequate material available were included in this study, without further selection. Patients diagnosed in 2005-2008, 2009-2014, 2015-2020 were enrolled in ALL-SCMC-2005 protocol, ALL-SCMC-2009 protocol and ALL-SCMC-2015 protocol respectively.
Ethics oversight	The study was approved by the Shanghai Children's Medical Center Institutional Review Board.

Note that full information on the approval of the study protocol must also be provided in the manuscript.

Field-specific reporting

Please select the one below that is the best fit for your research. If you are not sure, read the appropriate sections before making your selection.

☒ Life sciences ☐ Behavioural & social sciences ☐ Ecological, evolutionary & environmental sciences

For a reference copy of the document with all sections, see nature.com/documents/nr-reporting-summary-flat.pdf

Life sciences study design

All studies must disclose on these points even when the disclosure is negative.

Sample size	We analyzed 79 tumor samples from 61 relapsed B-ALL cases treated at Shanghai Children's Medical Center through 2007–2019, which have adequate tumor cells for ATAC-seq experiment. No sample size calculation was performed for the ATAC-seq, ChIP-seq and RNA-seq performed in this study.
Data exclusions	For comparison of OS and EFS between two treatment protocols (ALL-SCMC-2009 protocol and ALL-SCMC-2015 protocol), patients 118, 228, 273 and 284 with incomplete follow-up information and patients 155, 213, 289, 350 treated with ALL-SCMC-2005 protocol were excluded. For analysis of RFS-related ACRs, 4 samples (A424R, A485R, A429R and A357R) failed quality control and 4 samples (A155R, A213R, A289R, A350R) treated with ALL-SCMC-2005 protocol were excluded.
Replication	130 technical replicates were performed for 65 samples with adequate material. Pearson correlation coefficients between all technical replicates for each subtype indicate high reproducibility, with a median correlation coefficient of 0.9604 (ranging from 0.8850 to 0.9748).
Randomization	No randomization was performed in this study. Patients are grouped by B-ALL subtypes in the analysis for chromatin accessibility.
Blinding	Tumor types were blinded during generation of the sequencing data, including ATAC-seq, ChIP-seq and RNA-seq in this study. Investigators

Blinding | were blinded to the tumor type when processing the raw data and not blinded during the following analysis. One aim of this study was to discover the difference in chromatin accessibility between B-ALL subtype. So the analysis was performed comparing different subtypes.

Reporting for specific materials, systems and methods

We require information from authors about some types of materials, experimental systems and methods used in many studies. Here, indicate whether each material, system or method listed is relevant to your study. If you are not sure if a list item applies to your research, read the appropriate section before selecting a response.

Materials & experimental systems

n/a	Involved in the study	
☐	☒	Antibodies
☒	☐	Eukaryotic cell lines
☒	☐	Palaeontology and archaeology
☒	☐	Animals and other organisms
☒	☐	Clinical data
☒	☐	Dual use research of concern
☒	☐	Plants

Methods

n/a	Involved in the study	
☐	☒	ChIP-seq
☐	☒	Flow cytometry
☒	☐	MRI-based neuroimaging

Antibodies

Antibodies used

Antibodies for FACS:
anti-human CD19 conjugated with APC (Bioscience, catalog 17-0199-42, clone HIB19), anti-human CD10 conjugated with PECY7 (Biolegend, catalog 312214, clone HI10a), anti-human CD45 conjugated with APC-CY7 (Biolegend, catlaog 304014, clone HI30).
Above three antibodies were all used as 5ul/test in a final volume of 500 µL (1:100) for FACS staining.
Antibodies used for ChIP:
anti-histone H3K27ac antibody (Abcam, catalog 4729). For each sample, using 5ul antibody in 500ul volume for incubation with chromatin (1:100).

Validation

Validation of antibodies for the application and species are provided by the manufacturer.
human CD19 conjugated with APC: https://www.thermofisher.cn/order/genome-database/dataSheetPdf?producttype=antibody&productsubtype=antibody_primary&productId=17-0199-42&version=292;
human CD10 conjugated with PECY7: https://www.biolegend.com/en-us/products/pe-cyanine7-anti-human-cd10-antibody-5790?pdf=true&displayInline=true&leftRightMargin=15&topBottomMargin=15&filename=PE/Cyanine7%20anti-human%20CD10%20Antibody.pdf&v=20230114013553;
human CD45 conjugated with APC-CY7: https://www.biolegend.com/en-us/products/apc-cyanine7-anti-human-cd45-antibody-1914?pdf=true&displayInline=true&leftRightMargin=15&topBottomMargin=15&filename=APC/Cyanine7%20anti-human%20CD45%20Antibody.pdf&v=20221122052848
H3K27Ac: https://www.abcam.com/histone-h3-acetyl-k27-antibody-chip-grade-ab4729.pdf
Validation and relevant citations are included in above links.

Plants

Seed stocks

Report on the source of all seed stocks or other plant material used. If applicable, state the seed stock centre and catalogue number. If plant specimens were collected from the field, describe the collection location, date and sampling procedures.

Novel plant genotypes

Describe the methods by which all novel plant genotypes were produced. This includes those generated by transgenic approaches, gene editing, chemical/radiation-based mutagenesis and hybridization. For transgenic lines, describe the transformation method, the number of independent lines analyzed and the generation upon which experiments were performed. For gene-edited lines, describe the editor used, the endogenous sequence targeted for editing, the targeting guide RNA sequence (if applicable) and how the editor was applied.

Authentication

Describe any authentication procedures for each seed stock used or novel genotype generated. Describe any experiments used to assess the effect of a mutation and, where applicable, how potential secondary effects (e.g. second site T-DNA insertions, mosiacism, off-target gene editing) were examined.

ChIP-seq

Data deposition

☒ Confirm that both raw and final processed data have been deposited in a public database such as GEO.

☐ Confirm that you have deposited or provided access to graph files (e.g. BED files) for the called peaks.

Data access links
May remain private before publication.

Data are deposited in the Genome Sequence Archive (GSA) for Human of the National Genomics Data Center of China under accession number HRA002815 [https://ngdc.cncb.ac.cn/gsa-human/browse/HRA002815].

Files in database submission	C110D_Input, C110D_IP, C192D_Input, C192D_IP, C213R_H, C213R_IN, C226D_H, C226D_IN, C226R_H, C226R_IN, C233D_Input, C233D_IP, C262R, C262R_Input, C278D_Input, C278D_IP, C284R_Input, C284R_IP, C292R, C292R_Input, C390D_Input, C390D_IP, C451D_Input, C451D_IP
Genome browser session (e.g. UCSC)	Not applicable.

Methodology

Replicates	No replicate for ChIP-seq experiment in this study.
Sequencing depth	Paired-end 150bp length. The median number of total reads in 12 samples are 24397746 (experiment) and 24649343 (input); the median number of uniquely mapped reads in 12 samples are 17581182 (experiment) and 20444171 (input).
Antibodies	Human H3K27Ac antibody: ab4729, Abcam<br>Drosophila spike-in antibody: 61686, Active motif
Peak calling parameters	MACS2 parameters: -f BAMPE -g hs -B
Data quality	Input control was used to evaluate peak enrichment in MACS2 with a q cutoff value of 0.05.<br>The median number of peaks called in H3K27Ac ChIP-seq experiment for 12 samples was 53202 (ranging from 35558 to 93700).
Software	Burrows-Wheeler Aligner (v0.7.17-r1188) was used for mapping the clean reads to human genome (hg19). Uniquely mapped reads were extracted and marked duplicates with MarkDuplicates function in Picard (v2.22.9, http://broadinstitute.github.io/picard). MACS2 (v2.2.6) was used to call H3K27Ac modified regions of each sample.

Flow Cytometry

Plots

Confirm that:

☒ The axis labels state the marker and fluorochrome used (e.g. CD4-FITC).

☒ The axis scales are clearly visible. Include numbers along axes only for bottom left plot of group (a 'group' is an analysis of identical markers).

☒ All plots are contour plots with outliers or pseudocolor plots.

☒ A numerical value for number of cells or percentage (with statistics) is provided.

Methodology

Sample preparation	The cryopreserved leukemia cells were thawed in 37°C water bath. The LIVE/DEAD™ Fixable Dead Cell Stain Kits (Invitrogen, L23101), antibodies anti-human CD19 conjugated with APC (Bioscience,17-0199-42), anti-human CD10 conjugated with PECY7 (Biolegend, 312214) and anti-human CD45 conjugated with APC-CY7 (Biolegend, 304014) were used to enrich living tumor cells. Staining was performed for 30 minutes in dark at 4°C.
Instrument	FACS (Beckman, MoFlo XDP)
Software	FlowJo
Cell population abundance	The purity of samples was referred to the morphological examination and flow cytometric immunophenotyping of each patient at diagnosis (Supplementary Data 2).
Gating strategy	Preliminary FSC/SSC gates all cell population, and negative for LIVE/DEAD and positive for CD19/CD10 gates living B lineage cells. Among these cells, negative/dim for CD45 gates B-ALL leukemia cells.

☒ Tick this box to confirm that a figure exemplifying the gating strategy is provided in the Supplementary Information.
